# Handheld X-ray fluorescence geochemical data of geological and archaeological obsidian from Sonora, Mexico

**DOI:** 10.1016/j.dib.2020.106410

**Published:** 2020-10-22

**Authors:** Jesús Roberto Vidal-Solano, Alejandra Marisela Gómez-Valencia, Adriana Hinojo-Hinojo, Rufino Lozano-Santa Cruz

**Affiliations:** a Departamento de Geología, Universidad de Sonora (UNISON), Hermosillo, México; b Posgrado en Ciencias de la Tierra, Instituto de Geología, Estación Regional del Noroeste, Universidad Nacional Autónoma de México (ERNO-UNAM), Hermosillo, México; c Departamento de Ingeniería Civil y Minas, Universidad de Sonora (UNISON), Hermosillo, México; d Sección de Arqueología, Instituto Nacional de Antropología e Historia (Centro INAH Sonora) Hermosillo, México; e Laboratorio Nacional de Geoquímica y Mineralogía, Instituto de Geología, Universidad Nacional Autónoma de México (UNAM), Ciudad de México, México

**Keywords:** Obsidian, Energy-dispersive XRF, Geochemical data, NW Mexico, Archaeological artifacts

## Abstract

Geochemical detection using a portable XRF analyser is highly effective for nondestructive surface analysis in archaeological and geological obsidians. The data obtained in rock slabs, fragments, anhydrous nuclei, flaked and ground stone from Sonora, Mexico, were used to select certain analysed elements (Fe, Mn, Zr, Nb, Y, Th, Rb, Sr, Zn) that help to formulate geochemical variation diagrams to identify chemical trends and correlations between the samples. It proves to be an excellent analytical method for the provenance studies of archaeological artifacts. Subsequently, the integration of the obtained data here and their arrangement with the existing chemical analysis of obsidians for different localities of NW Mexico and the SW of the United States will allow for better knowledge of the primary sources of obsidian in the extraction and manufacture of archaeological artifacts**.**

## Specifications Table

**Table:** 

Subject	Geochemistry, Archaeology and Petrology
Specific subject area	Geoarchaeology (Obsidian provenance)
Type of data	Table (RAW DATA) Graphs Figure
How data were acquired	Determined by an X-Ray Fluorescence XL3t series NITON analyser from Thermo Fisher Scientific with support of the software Thermo Scientific™ Niton Data Transfer (NDT™)
Data format	Raw
Parameters for data collection	The analysis had an experimental stage to generate a final analytical protocol based on a repetition of at least three analyses per obsidian sample, procuring 120 counts per second, under the SOIL or TestAllGeo condition programs of Niton.
Description of data collection	The data was obtained from direct analysis on archaeological artifacts of obsidian of the Centro INAH-Sonora collections and geological materials of the La Rocateca-UNISON collection. Only some sections of rocks with relatively flat surfaces were required without any additional treatment of the geological samples. Excel was used to manage the database, descriptive analysis, and statistical evaluation were performed to define reliable element values.
Data source location	Institution: Servicio Geológico Mexicano (Gerencia Noroeste) City/Town/Region: Hermosillo, Sonora. Country: México
Data accessibility	Raw data in Mendeley Data https://data.mendeley.com/datasets/24kkg2hctg/5
Related research article	Co-submission: J.R. Vidal-Solano, A.M. Gómez-Valencia, A. Hinojo-Hinojo, R. Lozano-Santa Cruz., 2020. Geochemistry and geological control of Sonora obsidian: New insights into the provenance study of archaeological obsidians in Mexico, Journal of South American Earth Sciences, Volume 104, 102,840. https://doi.org/10.1016/j.jsames.2020.102840

## Value of the Data

• This dataset provides a chemical reference of the samples from primary obsidian sources reported so far in Sonora, for comparative processing in existing XRF databases.
• This dataset could be of interest to geologists studying the distribution and the characteristics of rhyolitic volcanic units in NW Mexico.
• This dataset could be of interest to archaeologists studying the obsidian provenance in Mexico and SW of the USA.
• Based on an XRF portable analyser application model, the geochemical data from various sets of archaeological artifacts could be of comparative interest to other specialists.

## 1 Data Description

**Fig. 1:**
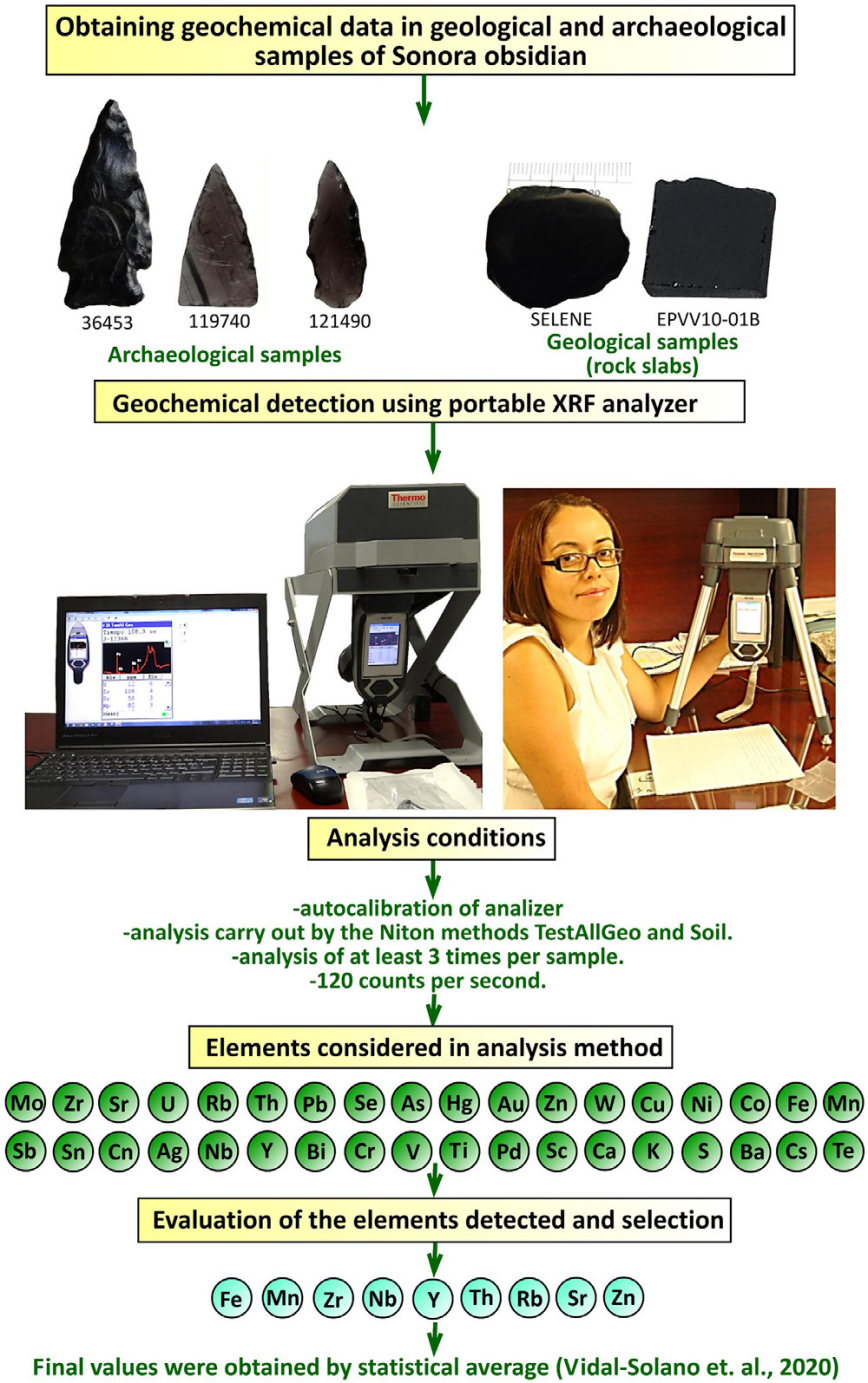
Analytical process concept map of the geological and archaeological specimens of obsidian from Sonora, Mexico.

**Fig. 2:**
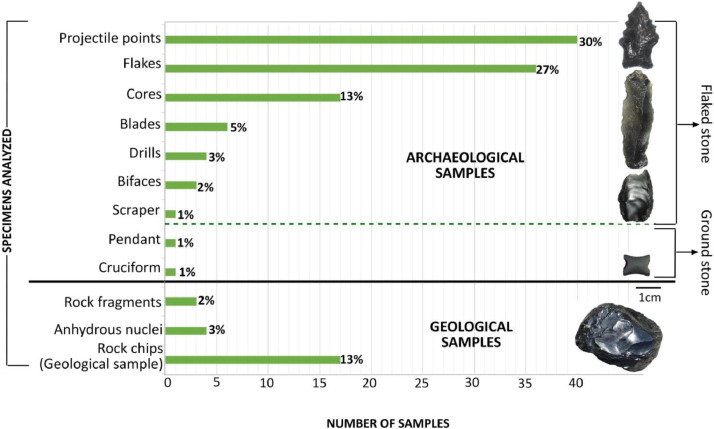
Graphical representation of the concentration and types of geological and archaeological specimens of obsidian from Sonora, Mexico, analysed by the dispersive energy XRF technique (Supplementary Data).

The raw geochemical data with Five hundred sixty-six analyses (Supplementary Data and Fig. 1), corresponding to 133 specimens of geological and archaeological obsidian artifacts mostly, including flaked and ground stone. Process 40 Projectile points, 36 varieties of lithic flakes, 17 lithic cores, 6 blades, 4 drills, 3 bifaces and 1 scraper were analysed; regarding ground stone objects, 1 cruciform and 1 pendant (Fig. 2) were analysed too. In the concerning of primary source geological samples, 17 rock slabs previously employed in the petrographic studies performed, 4 anhydrous nuclei and 3 obsidian fragments were also analysed (Fig. 2).

The analysed samples come from 13 archaeological sites: **1**, Caborca (SON:E:8:5); **2**, Trincheras (SON:F:11:37; SON:F:11:81; and SON:F:10:134); **3**, Hermosillo (SON:N:2:3); **4**, Sierra Libre (SON:O:5:15 and SON:O:5:14), **5**, Sierra Santa Úrsula (SON:O:13:4 and SON:O:13:5); **6**, Guaymas (SON:R:1:7; SON:N:16:6 and SON:N:16:8); **7**, Opodepe (SON:K:1:3); **8**, Cumpas (SON:L:1:6); **9**, Selene-Babidanchi (SON:H:16:3); **10**, Bacerac (CHIH:C:9:42); **11**, Bacerac el Gato (CHIH:C:14:3); **12**, Bavispe (CHIH:C:9:4); and **13**, Agua Prieta (SON:H:4:1, SON:H:4:2; SON:H:8:1 and SON:H:8:2). Geological primary obsidian sources localities comprise Vidrios Viejos and La Sierra Batamote at El Pinacate Region, Cerro Izabal at Hermosillo, Arroyo El Cajete and Arroyo El Galindro at Sierra Libre, and Arroyo San José de Robinson of the Ejido Francisco Villa at Sierra El Aguaje. The characteristics of these obsidian geological deposits and other new localities are described in [1].

## 2 Experimental Design, Materials and Methods

### 2.1 Selected samples

The initial stage in the obsidian analysis consisted of the petrological study of about 500 obsidian samples, both archaeological and geological, describing the physical parameters and appearance, the colour, the texture and the mineralogy to get the 133 obsidian specimens analysed [1] (Supplementary Data).

### 2.2 Sample processing and analysis

At the end of the experimental stage of the chemical detection of archaeological and geological obsidian samples, it was possible to establish an analysis protocol that was supported by the reliability values of the reference materials obtained by the portable analyser X-Ray Fluorescence XL3t (Fig. 1). The analysis method consisted first, in the auto-calibration of the device at the beginning of each examination stage, each measurement was 120 counts per second, and it was repeated at least three times per sample, slightly modifying the analysis position of the piece on each occasion. Subsequently, standardized reference materials [2], obsidian anhydrous nuclei and rock slabs were examined following the protocol to verify the reliability of the data. The device analysis mode was TestAllGeo or Soil method, which includes the widest element detection parameters to the geological application. The chemical elements considered by the *Niton Thermo Scientific, XL3t 500,* include: Mo, Zr, Sr, U, Rb, Th, Pb, Se, As, Hg, Au, Al, W, Cu, Ni, Co, Fe, Mn, Sb, Sn, Cd, Ag, Nb, Y, Bi, Cr, V, Ti, Pd, Sc, Ca, K, S, Ba, Cs y Te. The raw data of X-Ray analyses are presented in the supplementary material at Mendeley Data [https://data.mendeley.com/datasets/24kkg2hctg/5].

**Fig. 3:**
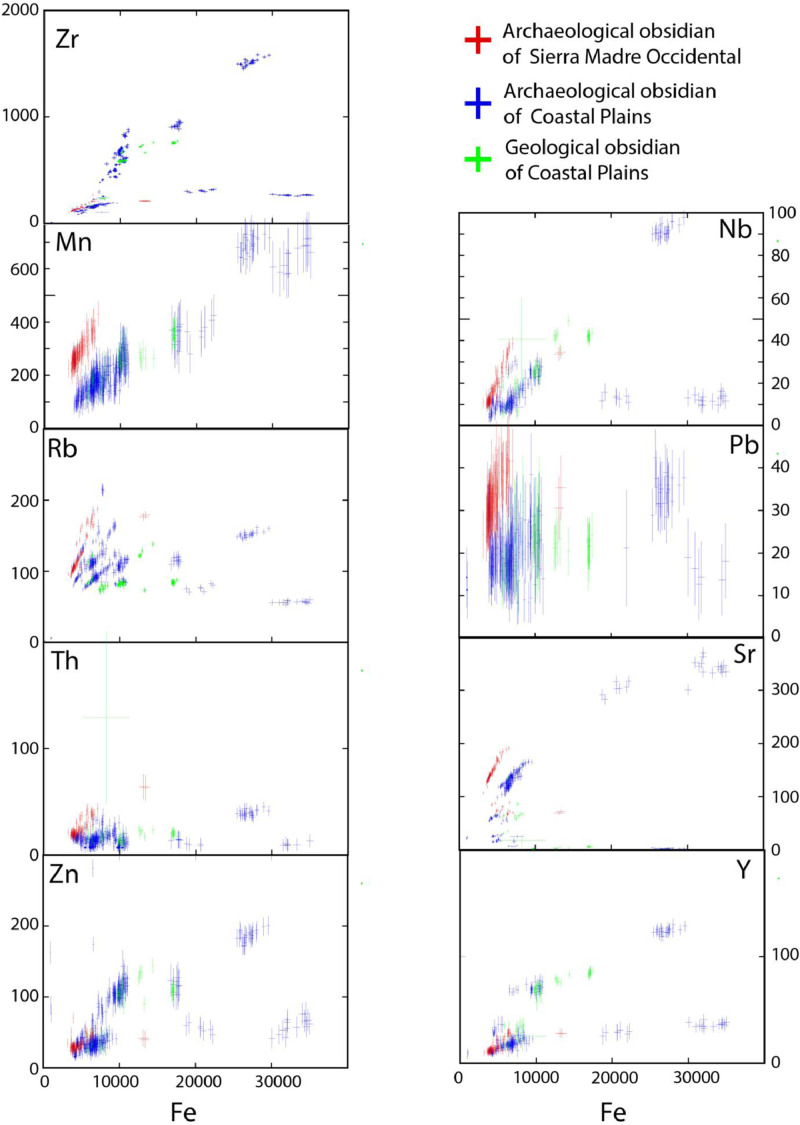
Geochemical variation diagrams for the elements selected and their analytical error in the ED- XRF raw data of the geological and archaeological specimens of obsidian analysed from Sonora, Mexico. The main chemical characteristics of obsidian samples related to geographical provinces can be observed.

Subsequently, data processing was performed in Excel, which consisted of evaluating values obtained and its relationship whit the analytical error for each element concerning the reference standard. Elements with a low detection limit and those with high errors were discarded. The study obtained reliable detection values for Zr, Sr, Rb, Th, Pb, Zn, Fe, Mn, Nb and Y (Fig 1 and Fig. 3). Based on these chemical element values, a statistical average and a confidence limit at 95% were calculated using the student T for the number of measures in each sample. Some specimens are not reported because the measurements obtained were below the detection value [3] for certain chemical elements, or they were eventually measured at several times less than that required for the reported statistical values. Fig. 3 shows the variability and representativeness of the content of the nine selected elements, with their analytical error, for the geological and archaeological obsidian analyses, and the relations according to the geographical provenance. Good definitions and a tendency in the variation of the contents can be observed in most elements except Pb. In addition, the archaeological samples from one province (Coastal Plains of Sonora, CPS) have a wide range in the element contents such as Mn, Rb, Zn, Nb, Sr and Y, but especially in Fe and Zr, with values from 3000 ppm to more than 30,000 ppm and from ∼100 ppm to ∼1500 ppm respectively. The chemical contents of geological obsidian samples from CPS sources are consistent with some values of the archaeological samples from the same province.

## Ethics Statement

Does not apply to this data.

## CRediT authorship contribution statement

**Jesús Roberto Vidal-Solano:** Conceptualization, Methodology, Investigation, Writing - original draft, Writing - review & editing, Supervision. **Alejandra Marisela Gómez-Valencia:** Validation, Formal analysis, Data curation, Writing - original draft, Writing - review & editing, Visualization, Project administration. **Adriana Hinojo-Hinojo:** Validation, Formal analysis, Resources, Data curation, Writing - original draft, Writing - review & editing, Visualization. **Rufino Lozano-Santa Cruz:** Validation, Investigation, Resources, Writing - review & editing.

## Declaration of Competing Interest

The authors declare that they have no known competing financial interests or personal relationships which have, or could be perceived to have, influenced the work reported in this article.

## Supplementary Data

Raw data to this article can be found online at Mendeley Data https://data.mendeley.com/datasets/24kkg2hctg/5 or http://dx.doi.org/10.17632/24kkg2hctg.5
